# 

**Published:** 1863-04

**Authors:** 


### THE MEDICAL CRITIC AND PSYCHOLOGICAL JOURNAL.

EDITED BY
FORBES WINSLOW, M.D., D.C.L. Oxon.

No. X.—APRIL, 1863.

TO BE CONTINUED QUARTERLY.

LONDON:
JOHN W. DAVIES, 54, PRINCES STREET,
LEICESTER SQUARE.
EDINBURGH: MACLACHLAN AND CO. DUBLIN: FANNIN AND CO.

*Price Three Shillings and Sixpence.*

SAVILL AND EDWARDS,] [4, CHANDOS STREET.

